# Analysis of the Long-Term Impact on Cellular Immunity in COVID-19-Recovered Individuals Reveals a Profound NKT Cell Impairment

**DOI:** 10.1128/mBio.00085-21

**Published:** 2021-04-27

**Authors:** Jia Liu, Xuecheng Yang, Hua Wang, Ziwei Li, Hui Deng, Jing Liu, Shue Xiong, Junyi He, Xuemei Feng, Chunxia Guo, Weixian Wang, Gennadiy Zelinskyy, Mirko Trilling, Kathrin Sutter, Tina Senff, Christopher Menne, Joerg Timm, Yanfang Zhang, Fei Deng, Yinping Lu, Jun Wu, Mengji Lu, Dongliang Yang, Ulf Dittmer, Baoju Wang, Xin Zheng

**Affiliations:** a Department of Infectious Diseases, Union Hospital, Tongji Medical College, Huazhong University of Science and Technology, Wuhan, China; b Institute for Virology, University Hospital of Essen, University of Duisburg-Essen, Essen, Germany; c Joint International Laboratory of Infection and Immunity, Huazhong University of Science and Technology, Wuhan, China; d Institute for Virology, Heinrich-Heine-University, University Hospital, Duesseldorf, Germany; e Wuhan Institute of Virology, Chinese Academy of Sciences, Wuhan, China; Johns Hopkins Bloomberg School of Public Health

**Keywords:** COVID-19, NKT cell, SARS-CoV-2, cellular immunity

## Abstract

The coronavirus disease 2019 (COVID-19) pandemic caused by the severe acute respiratory syndrome coronavirus 2 (SARS-CoV-2) affected over 120 million people and killed over 2.7 million individuals by March 2021. While acute and intermediate interactions between SARS-CoV-2 and the immune system have been studied extensively, long-term impacts on the cellular immune system remain to be analyzed. Here, we comprehensively characterized immunological changes in peripheral blood mononuclear cells in 49 COVID-19-convalescent individuals (CI) in comparison to 27 matched SARS-CoV-2-unexposed individuals (UI). Despite recovery from the disease for more than 2 months, CI showed significant decreases in frequencies of invariant NKT and NKT-like cells compared to UI. Concomitant with the decrease in NKT-like cells, an increase in the percentage of annexin V and 7-aminoactinomycin D (7-AAD) double-positive NKT-like cells was detected, suggesting that the reduction in NKT-like cells results from cell death months after recovery. Significant increases in regulatory T cell frequencies and TIM-3 expression on CD4 and CD8 T cells were also observed in CI, while the cytotoxic potential of T cells and NKT-like cells, defined by granzyme B (GzmB) expression, was significantly diminished. However, both CD4 and CD8 T cells of CI showed increased Ki67 expression and were fully able to proliferate and produce effector cytokines upon T cell receptor (TCR) stimulation. Collectively, we provide a comprehensive characterization of immune signatures in patients recovering from SARS-CoV-2 infection, suggesting that the cellular immune system of COVID-19 patients is still under a sustained influence even months after the recovery from disease.

## Abstract (precis)

Wuhan was the very first city hit by SARS-CoV-2. Accordingly, the patients who experienced the longest phase of convalescence following COVID-19 reside here.

## INTRODUCTION

The sudden emergence and rapid global spread of severe acute respiratory syndrome coronavirus 2 (SARS-CoV-2) and the resulting coronavirus disease 2019 (COVID-19) pose an unprecedented health crisis to humankind. As of 21 March 2021, there were about 122 million documented cases of SARS-CoV-2 infection and more than 2.7 million individuals had lost their lives. SARS-CoV-2-infected people exhibit a wide spectrum of disease manifestations ranging from moderate or even unnoticed symptoms (1) to life-threatening acute infections predominantly affecting the respiratory tract (2), but also other organs such as the kidney (3) and the central nervous system (4) can be harmed. Moderate cases show symptoms of fever, dry cough, fatigue, and abnormal chest computed tomography (CT) findings but with a good prognosis (5, 6). Conversely, some patients suddenly deteriorate toward acute respiratory distress syndrome (ARDS) or multiple organ failure, with fatality rates approaching 60% (7).

Recent studies demonstrated that SARS-CoV-2 infections strongly shape the immune system and result in its dysregulation, including imbalanced antiviral and proinflammatory responses and altered numbers and impaired functions of different immune cell subsets (8). We and others previously showed that lymphopenia and an inflammatory cytokine storm can be observed in COVID-19 patients and that their extents correlate with COVID-19-associated disease severity and mortality (9–12). The recovery of T cell counts and the end of the inflammatory cytokine storm in severe COVID-19 cases have been associated with a favorable disease outcome (9). However, the impact of SARS-CoV-2 on the cellular immune system after the recovery from the disease in the long term remains largely unknown.

Applying multicolor flow cytometry, we comprehensively characterized immunological changes in peripheral blood mononuclear cells (PBMCs) in 49 convalescent SARS-CoV-2-infected individuals (CI) in comparison to 27 matched SARS-CoV-2-unexposed individuals (UI). Our data suggest that the immune system remains heavily influenced months after resolving SARS-CoV-2 infection.

## RESULTS

### Characteristics of the study cohort.

To characterize the cellular immune system in individuals who had recovered from COVID-19, blood samples were analyzed about 3.5 months (Chinese cohort) or 1.5 months (German cohort) after the first diagnosis. The demographic profiles of the Chinese cohort are shown in Table 1. All CI had been diagnosed as either moderate (83.3%, 25/30) or mild (16.7%, 5/30) COVID-19 cases. The median period between the first diagnosis of COVID-19 and blood sampling was 112 days (range: 60 to 136 days). Among all COVID-19 cases, 43.3% (13/30) were hospitalized and 23.3% (7/30) received oxygen inhalation treatment. Leukopenia and lymphopenia were observed in 43.5% (10/23) and 60.9% (14/23) of tested cases, respectively. Increased C-reactive protein and interleukin-6 (IL-6) levels were observed in 52.6% (10/19) and 76.9% (10/13) of tested patients, respectively. All moderate cases showed abnormal radiological findings suggesting pneumonia by chest computed tomography (CT) scans, while mild cases showed no radiological abnormality in the lungs. Twelve moderate cases and one mild case (43.3%, 13/30) had positive RT-PCR results for viral RNA. All patients were confirmed anti-SARS-CoV-2 IgM and IgG seropositive. At the time of blood sampling, 20% (6/30) of cases exhibited virus-specific IgM and IgG, 70% (21/30) were IgG single positive, and 10% (3/30) were IgM and IgG negative. The demographic profiles of the German cohort are shown in Table 2. This cohort has 19 CI, in which 10.53% (2/19) were hospitalized. Nine cases (47.37%, 9/19) had positive RT-PCR results for viral RNA, and 18 cases (94.74%, 18/19) were anti-SARS-CoV-2 IgA and/or IgG seropositive. The median period between the first diagnosis of COVID-19 and blood sampling in the German cohort was 41 days. Only the analysis of invariant NKT (iNKT) cells was performed in the German cohort, while analysis of all other cell populations was performed in the Chinese cohort.

**TABLE 1:** Baseline characteristics of the Chinese cohort^*a*^

Parameter	Unexposed individuals	Convalescent individuals
*n*	21	30
Gender (M/F)	3/18	3/27
Age, yr	33.5	36.8
Mild cases, %	/	16.67% (5/30)
Moderate cases, %	/	83.33% (25/30)
Days from diagnosis	/	112 (60–136)
Clinical parameters, %		
Fever	/	30.00% (9/30)
Respiratory symptoms	/	43.33% (13/30)
Hospitalized	/	43.33% (13/30)
Oxygen therapy	/	23.33% (7/30)
Laboratory parameters, %		
Leukopenia	/	43.48% (10/23)
Lymphopenia	/	60.87% (14/23)
Increased CRP	/	52.63% (10/19)
Increased ferritin	/	12.50% (1/8）
Increased LDH	/	14.29% (2/14)
Abnormal liver function	/	42.86% (6/14)
Abnormal renal function	/	0% (0/14)
Increased CK	/	7.69% (1/13）
Abnormal blood coagulation	/	38.46% (5/13)
Increased IL-6	/	76.92 (10/13)
CT scan, %		
Normal	/	16.67% (5/30)
Viral pneumonia	/	83.33% (25/30)
Virological markers		
RNA positive, %	/	43.33% (13/30)
Time of primary PCR testing (mean)		1–53 dpo (11.3)
IgM single positive, %	/	0.00% (0/30)
IgG single positive, %	/	80.00% (24/30)
IgM and IgG positive, %	/	20.00% (6/30)
IgG turning negative, %	/	10% (3/30)
Neutralizing antibody, %	/	90% (27/30)

a Abbreviations: dpo, days post-disease onset; CRP, C-reactive protein; LDH, lactate dehydrogenase; CK, creatine kinase. /, not applicable.

**TABLE 2:** Baseline characteristics of the German cohort

Parameter	Unexposed individuals	Convalescent individuals
*n*	6	19
Gender (M/F)	2/4	10/9
Age, yr (mean)	46.3	44.8
Days from diagnosis	/	41 (14–81)
Hospitalized, %	/	10.53% (2/19)
RNA positive	/	47.37% (9/19)
Time of primary PCR testing (mean)		−2–11 dpo (5.5)
Anti-SARS-CoV-2 IgA and/or IgG positive^*a*^	/	94.74% (18/19)

a Optical density (OD) ratio ≥1.1 for IgA and IgG was considered positive (Euroimmun enzyme-linked immunosorbent assay [ELISA]); index (sample/cut-off [S/C]) of ≥1.4 is considered positive (Abbott CMIA); dpo, days post-disease onset. /, not applicable.

The criteria for COVID-19 convalescence are as follows: afebrile for more than 3 days, resolution of respiratory symptoms, substantial improvement of chest CT images, and two consecutive negative RT-qPCR tests for viral RNA in respiratory tract swab samples obtained at least 24 h apart. At time of blood sampling, all CI were negative for viral RNA test and had no recognized medical conditions.

### Characterization of immune cell subsets in individuals recovering from COVID-19.

**FIG 1:**
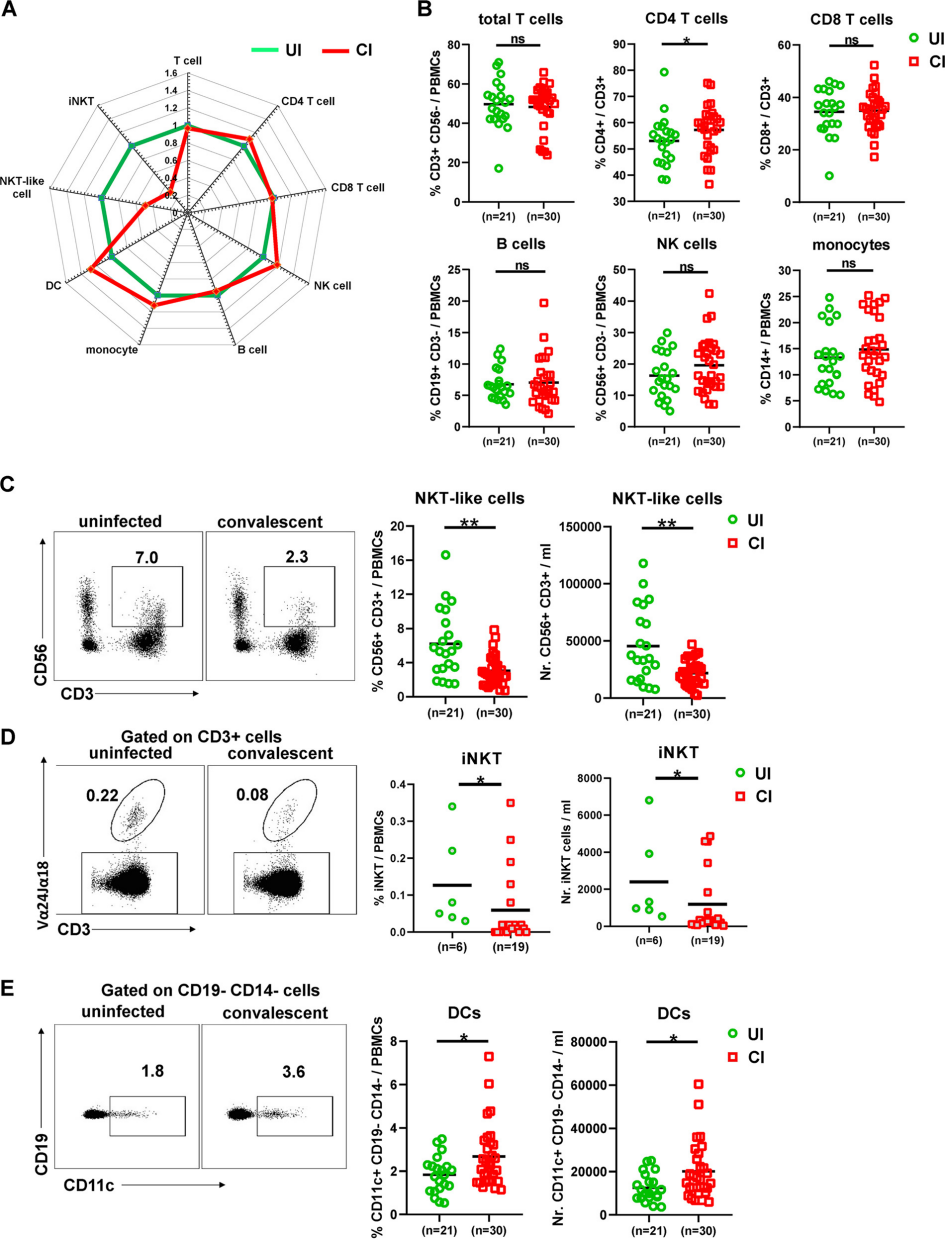
Characterization of immune cell subsets in individuals recovering from COVID-19. (A) The fold changes of the percentages (median) of total T cells, CD4 and CD8 T cells, B cells, NK cells, NKT-like cells, dendritic cells, and monocytes in the blood of CI (*n* = 30) compared to those of UI (*n* = 21) are depicted by radar plots. (B) The percentages of total T cells, CD4 and CD8 T cells, B cells, NK cells, and monocytes in the blood of UI (*n* = 21) and CI (*n* = 30) were analyzed by flow cytometry. (C) The absolute numbers and percentages of NKT-like cells in the blood of UI (*n* = 21) and CI (*n* = 30) were analyzed by flow cytometry. (D) The absolute numbers and percentages of invariant NKT (iNKT) cells in the blood of UI (*n* = 6) and CI (*n* = 19) were analyzed by flow cytometry. (E) The absolute numbers and percentages of dendritic cells in the blood of UI (*n* = 21) and CI (*n* = 30) were analyzed by flow cytometry. CI, COVID-19-convalescent individuals; UI, SARS-CoV-2-unexposed individuals. Statistically significant differences are indicated by asterisks (*, <0.05; **, <0.01; nonparametric Mann-Whitney test).

First, we characterized whether the overall immune cell composition in PBMCs differs between CI and UI by flow cytometry (as depicted in Fig. S1A and B in the supplemental material). We observed that the profile of the immune cell composition of CI was distinct from that of UI (Fig. 1A). Specifically, the frequencies but not the absolute numbers of CD4^+^ T cells in CI were slightly but significantly higher than in UI (Fig. 1B and Fig. S1C), while no significant differences in absolute numbers and frequencies of total T cells, CD8^+^ T cells, B cells, NK cells, and monocytes were observed between CI and UI (Fig. 1B and Fig. S1C). Interestingly, CI showed dramatic decreases in absolute numbers and frequencies of both the NKT-like cell population (CD3^+^ CD56^+^) and the iNKT cell population (CD3^+^ and T cell receptor [TCR] Vα24-Jα18^+^) compared to UI (Fig. 1C and D). The absolute numbers of NKT-like cells of CI (median: 20.7/μl) were only about 60% of the level observed in UI (median: 34.5/μl, Fig. 1C). Besides, CI showed a significant increase in dendritic cells (DCs) in both the absolute numbers and frequencies compared to UI (Fig. 1E).

10.1128/mBio.00085-21.1 FIG S1 Characterization of immune cell subset profiles in the PBMCs of individuals recovering from COVID-19. (A) Exemplary gating strategy for definition of T cells, NK cells, NKT-like cells, B cells, dendritic cells, and monocytes by flow cytometry. (B) Exemplary gating strategy for definition of invariant NKT (iNKT) cells by flow cytometry. (C) The absolute numbers of total T cells, CD4 and CD8 T cells, B cells, NK cells, and monocytes in the blood of UI (*n* = 21) and CI (*n* = 30) were analyzed by flow cytometry. CI, COVID-19-convalescent individuals; UI, SARS-CoV-2-unexposed individuals; ns, not significant (*P* > 0.05). Download FIG S1, TIF file, 2.3 MB. Copyright © 2021 Liu et al. 2021 Liu et al. https://creativecommons.org/licenses/by/4.0/ This content is distributed under the terms of the Creative Commons Attribution 4.0 International license.

**FIG 2:**
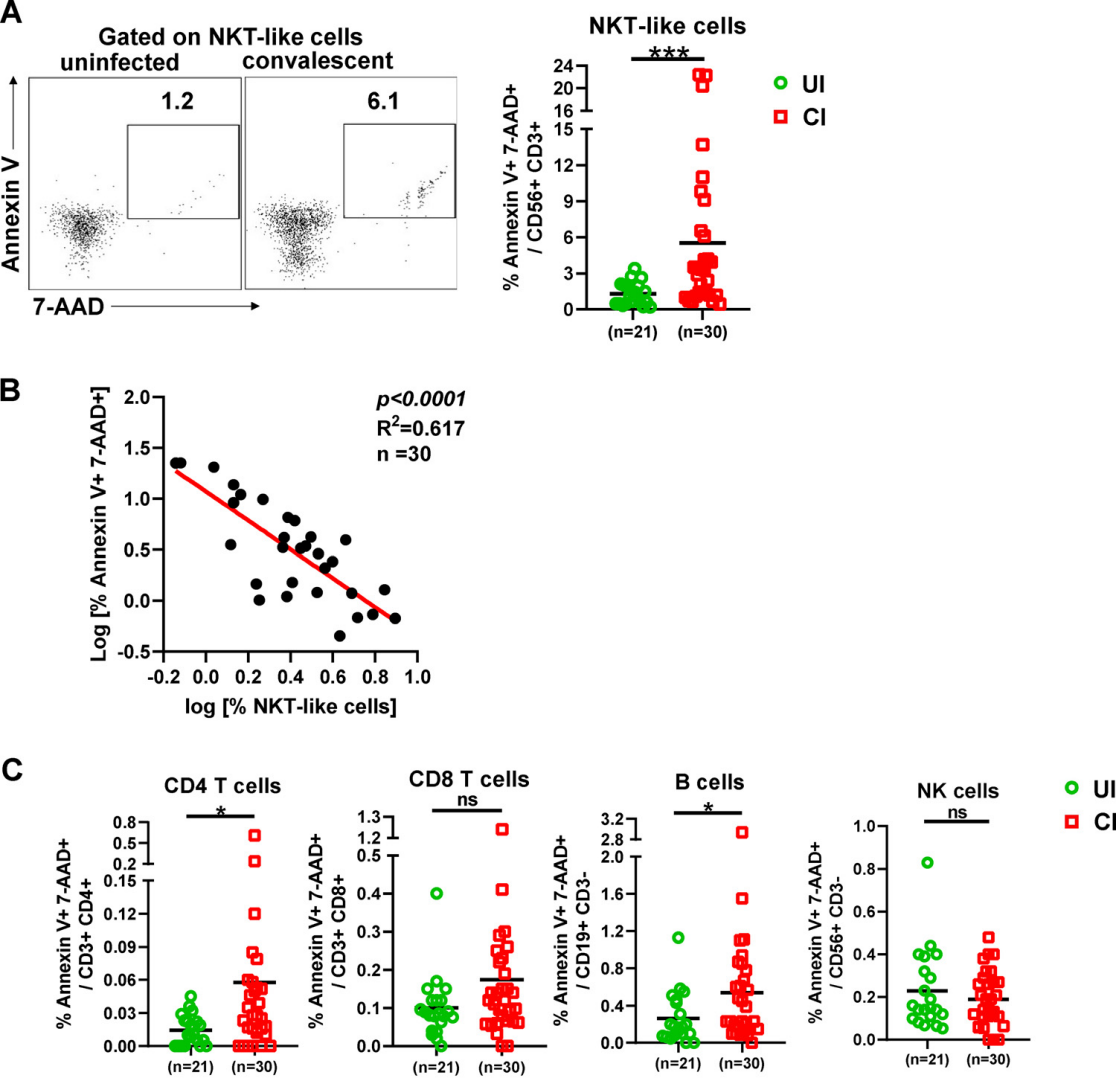
Characterization of immune cell death in individuals recovering from COVID-19. (A) The percentages of early apoptosis (annexin V^+^ 7-AAD^−^) and late apoptosis/necroptosis (annexin V^+^ 7-AAD^+^) of NKT-like cells in the blood of CI (*n* = 30) compared to those of UI (*n* = 21) were analyzed by flow cytometry. (B) Correlation analysis between the frequencies and the late apoptosis/necroptosis of NKT-like cells was performed in CI. (C) The percentages of early apoptosis (annexin V^+^ 7-AAD^−^) and late apoptosis/necroptosis (annexin V^+^ 7-AAD^+^) of CD4 T, CD8 T, B, and NK cells in the blood of CI (*n* = 30) compared to those of UI (*n* = 21) were analyzed by flow cytometry. CI, COVID-19-convalescent individuals; UI, SARS-CoV-2-unexposed individuals. Statistically significant differences are indicated by asterisks (*, <0.05; **, <0.01; ns, not significant; nonparametric Mann-Whitney test).

Next, we examined whether the decrease of NKT-like cells in CI was associated with increased cell death. Annexin V and 7-aminoactinomycin D (7-AAD) stainings were performed to analyze apoptosis and necroptosis of NKT-like cells, CD4 and CD8 T cells, B cells, and NK cells. A profound and significant increase in the frequency of the annexin V and 7-AAD double-positive NKT-like cells was observed in CI (median: 3.3%) compared to UI (median: 1.2%) (Fig. 2A), suggesting that increased proportions of NKT-like cells of CI are undergoing apoptosis and/or necroptosis even after recovery from COVID-19. Importantly, the intensities of NKT-like cell death were inversely correlated with NKT cell frequencies in CI (Fig. 2B). No significant correlations were observed between either the frequencies or cell death of NKT-like cells and the days post-disease onset (Fig. S2A). Besides, apoptosis and/or necroptosis of CD4^+^ T cells and B cells was also slightly increased in CI compared to those in UI (Fig. 2C).

10.1128/mBio.00085-21.2 FIG S2 Correlation between the changes of cellular immunity and the time that had elapsed from disease onset. (A) The correlation of the frequencies and cell death of NKT-like cells with the days post-disease onset are shown. (B) The correlation of the granzyme B expression of CD8 T cells, CD4 T cells, and NKT-like cells with days post-disease onset are shown. The Pearson product-moment correlation coefficient test was used to test the significance, and *P* value and *r*^2^ value (correlation coefficient) are indicated in each panel. Download FIG S2, TIF file, 0.3 MB. Copyright © 2021 Liu et al. 2021 Liu et al. https://creativecommons.org/licenses/by/4.0/ This content is distributed under the terms of the Creative Commons Attribution 4.0 International license.

Taken together, these results demonstrate that SARS-CoV-2 infections elicit a sustained impact on the immune cell composition in the peripheral blood during the extended convalescence phase, dominated by a contraction of NKT-like cells and an expansion of DCs.

### Characterization of T cell phenotypes in individuals recovering from COVID-19.

**FIG 3:**
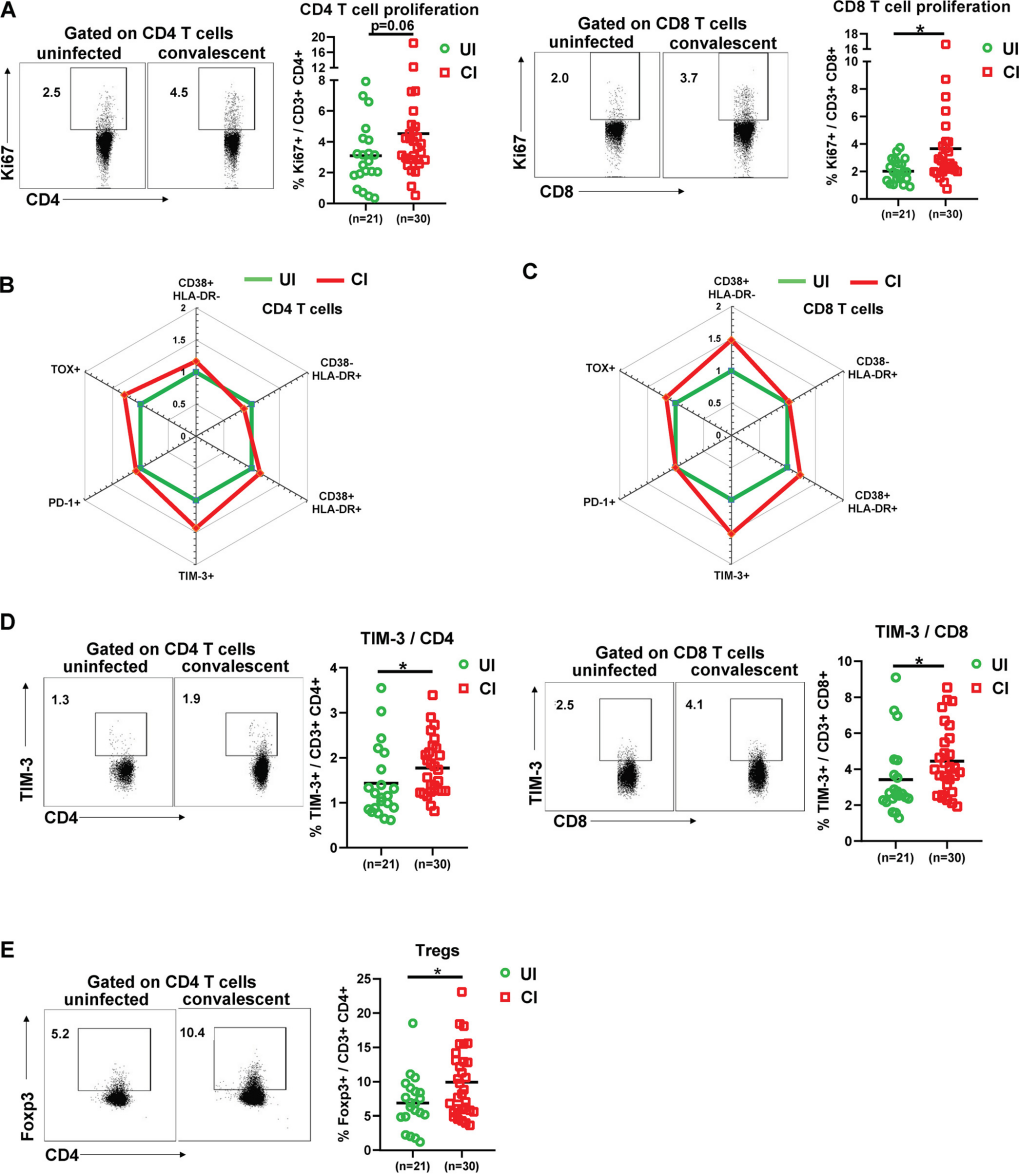
Characterization of T cell phenotypes in the PBMCs of individuals recovering from COVID-19. (A) The percentages of Ki67^+^ CD4 and CD8 T cells in the blood of UI (*n* = 21) and CI (*n* = 30) were analyzed by flow cytometry. (B and C) The fold changes of the percentages (median) of CD38^+^ HLA-DR^−^, CD38^−^ HLA-DR^+^, CD38^+^ HLA-DR^+^, PD-1^+^ TOX^−^, PD-1^−^ TOX^+^, PD-1^+^ TOX^+^, and TIM-3^+^ CD4 and CD8 T cells in the blood of CI (*n* = 30) compared to those of UI (*n* = 21) are depicted by radar plots. (D) The percentages of TIM-3^+^ CD4 and CD8 T cells in the blood of UI (*n* = 21) and CI (*n* = 30) were analyzed by flow cytometry. (E) The percentages of Foxp3^+^ CD4 T cells in the blood of UI (*n* = 21) and CI (*n* = 30) were analyzed by flow cytometry. CI, COVID-19-convalescent individuals; UI, SARS-CoV-2-unexposed individuals. Statistically significant differences are indicated by asterisks (*, <0.05; **, <0.01; nonparametric Mann-Whitney test).

Next, we used several markers of CD4 and CD8 T cells to determine their differentiation (CD45RA and CCR7), proliferation (Ki67), activation (CD38 and HLA-DR), and exhaustion/suppression (PD-1, TIM-3, TOX, and regulatory T cells [Tregs]) status. We also defined different subpopulations, and T cells from UI and CI were divided into naive (CD45RA^+^ CCR7^+^), central memory (TCM, CD45RA^−^ CCR7^+^), effector memory (TEM, CD45RA^−^ CCR7^−^), and terminally differentiated effector (TEMRA, CD45RA^+^ CCR7^−^) subpopulations (Fig. S3). No significant differences were observed between UI and CI for any of the CD4 or CD8 T cell subpopulations mentioned above, although a tendency of decreased CD4^+^ TEMRA cell frequencies in CI (median: 3.3%) compared to UI (median: 7.5%) was observed (Fig. S3). The proliferation (Ki67^+^) expression of both CD4 and CD8 T cells in CI was higher than that in UI (median: CD4 3.8% versus 2.7%, CD8 2.5% versus 1.9%), and the difference for CD8 T cells was statistically significant (Fig. 3A), indicating that T cells from CI show an enhanced proliferation capacity. Previous studies have shown that T cells are highly activated during the acute phase of COVID-19 (13); thus, we next analyzed the activation status of CD4 and CD8 T cells by examining CD38 and HLA-DR expression on the cell surface. No significant differences in CD38 and HLA-DR expression on CD4 T cells were observed between UI and CI (Fig. 3B and Fig. S4A). Compared to UI, CI showed a 1.47-fold increase in the frequencies of CD38^+^ HLA-DR^−^ CD8 T cells; however, this difference was not statistically significant (Fig. 3C and Fig. S4A). Based on the analysis of PD-1 expression, some studies reported that CD8 T cells may already become functionally exhausted during the acute phase of COVID-19 (14), which was questioned by a different study from our group (15). In our current study, the PD-1 expression levels on CD4 or CD8 T cells from CI were similar to those in UI (Fig. 3B and C and Fig. S3B). However, the expression of TIM-3, another immune checkpoint molecule, was increased about 20% on CD4 and CD8 T cells in CI compared to UI, and this difference was statistically significant (Fig. 3D). Moreover, we examined the expression of TOX in T cells from our study subjects, which is a newly identified key factor of T cell exhaustion (16, 17). The frequencies of TOX^+^ CD4 and CD8 T cells in CI increased around 20 to 30% compared to those in UI; however, the differences were not statistically significant (Fig. 3B and C and Fig. S4B). Regulatory T cells (Tregs) play a very important role in controlling immunopathogenic reactions upon infections by dampening pathogen-specific immune responses (18–20). We therefore examined the frequencies of Tregs in the PBMCs of CI by analyzing Foxp3 expression in CD4 T cells. As shown in Fig. 3E, CI showed a significant increase in Treg frequencies (median: 8.8%) compared to those in UI (median: 6.8%).

10.1128/mBio.00085-21.3 FIG S3 Characterization of T cell differentiation in the PBMCs of individuals recovering from COVID-19. The percentages of naive (CD45RA^+^ CCR7^+^), central memory (TCM, CD45RA^−^ CCR7^+^), effector memory (TEM, CD45RA^−^ CCR7^−^), and terminally differentiated effector memory (TEMRA, CD45RA^+^ CCR7^−^) CD4 and CD8 T cells in the blood of UI (*n* = 21) and CI (*n* = 30) were analyzed by flow cytometry. CI, COVID-19-convalescent individuals; UI, SARS-CoV-2-unexposed individuals; ns, not significant (*P* > 0.05). Download FIG S3, TIF file, 0.8 MB. Copyright © 2021 Liu et al. 2021 Liu et al. https://creativecommons.org/licenses/by/4.0/ This content is distributed under the terms of the Creative Commons Attribution 4.0 International license.

10.1128/mBio.00085-21.4 FIG S4 Characterization of T cell phenotypes in the PBMCs of individuals recovering from COVID-19. (A) The percentages of CD38^+^ HLA-DR^−^, CD38^−^ HLA-DR^+^, and CD38^+^ HLA-DR^+^ CD4 and CD8 T cells in the blood of CI (*n* = 30) compared to those of UI (*n* = 21) were analyzed by flow cytometry. (B) The percentages of PD-1^+^ and TOX^+^ CD4 and CD8 T cells in the blood of CI (*n* = 30) compared to those of UI (*n* = 21) were analyzed by flow cytometry. CI, COVID-19-convalescent individuals; UI, SARS-CoV-2-unexposed individuals; ns, not significant (*P* > 0.05). Download FIG S4, TIF file, 0.8 MB. Copyright © 2021 Liu et al. 2021 Liu et al. https://creativecommons.org/licenses/by/4.0/ This content is distributed under the terms of the Creative Commons Attribution 4.0 International license.

Taken together, our results demonstrate an immune environment that is prone toward T cell suppression during the late COVID-19 convalescent phase. However, T cells in CI still show a slightly enhanced activation and proliferation status, suggesting that these individuals are situated in a phase of ongoing restoration of the immune homeostasis.

### Characterization of cytotoxic effector profiles of T, NK, and NKT-like cells in individuals recovering from COVID-19.

**FIG 4:**
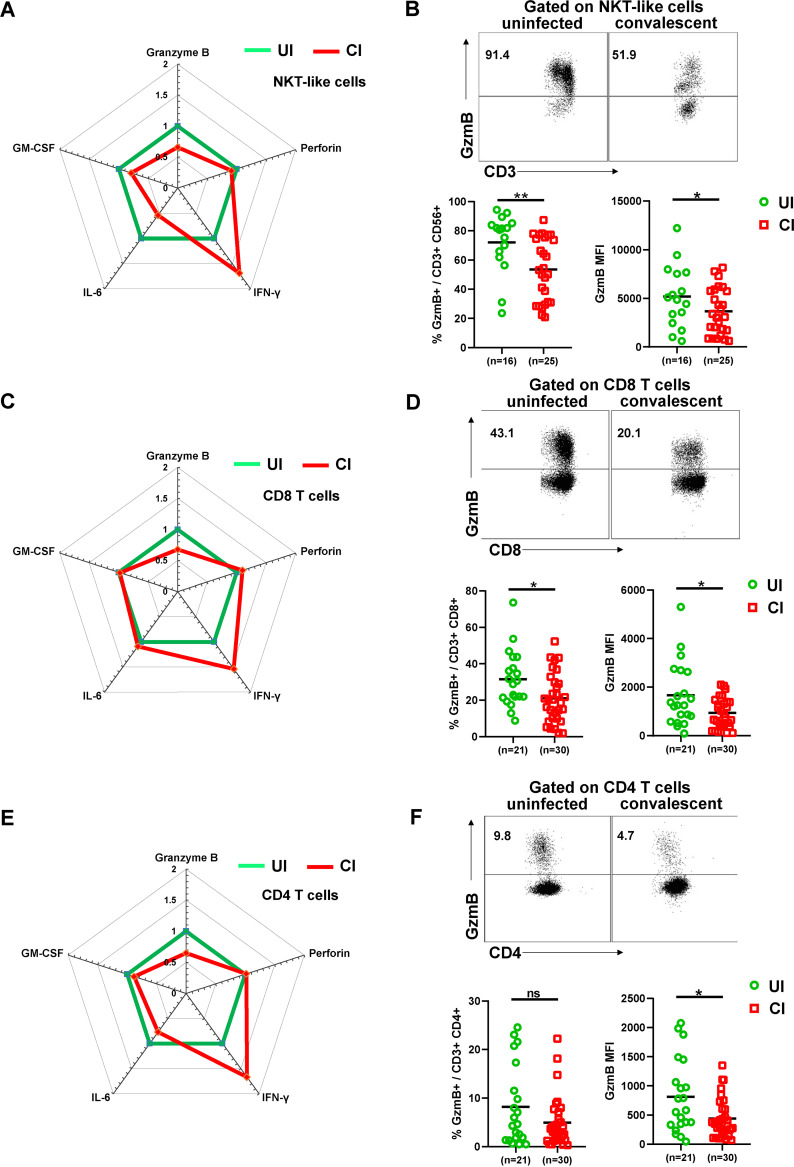
Characterization of cytotoxic and cytokine profiles of T, NK, and NKT-like cells in the PBMCs of individuals recovering from COVID-19. (A) The fold changes of the percentages (median) of granzyme B-, perforin-, IFN-γ-, IL-6-, and GM-CSF-producing NKT-like cells in the blood of CI compared to those of UI are depicted by radar plots. (B) The percentages and MFI (geometric mean) of granzyme B expression of NKT-like cells in the blood of UI (*n* = 16) and CI (*n* = 25) were analyzed by flow cytometry. (C) The fold changes of the percentages (median) of granzyme B-, perforin-, IFN-γ-, IL-6-, and GM-CSF-producing CD8 T cells in the blood of CI compared to those of UI are depicted by radar plots. (D) The percentages and MFI (geometric mean) of granzyme B expression of CD8 T cells in the blood of UI (*n* = 21) and CI (*n* = 30) were analyzed by flow cytometry. (E) The fold changes of the percentages (median) of granzyme B-, perforin-, IFN-γ-, IL-6-, and GM-CSF-producing CD4 T cells in the blood of CI compared to those of UI are depicted by radar plots. (F) The percentages and MFI (geometric mean) of granzyme B expression of CD4 T cells in the blood of UI (*n* = 21) and CI (*n* = 30) were analyzed by flow cytometry. CI, COVID-19-convalescent individuals; UI, SARS-CoV-2-unexposed individuals. Statistically significant differences are indicated by asterisks (*, <0.05; **, <0.01; nonparametric Mann-Whitney test).

To characterize their cytotoxic profiles, we intracellularly stained CD4, CD8, NKT-like, and NK cells for the cytotoxic molecules granzyme B (GzmB) and perforin directly *ex vivo* without restimulation and compared UI with CI. CI showed significant decreases in the frequencies of GzmB-producing NKT-like cells (median: 53.2%) and CD8 T cells (mean: 20.3%) compared to UI (NKT-like cells: 81.2%, CD8 T cells: 29.8%; Fig. 4A to D). A tendency of decreased frequencies of GzmB-producing CD4 T cells was also observed; however, this difference was not statistically significant (Fig. 4E and F). Consistently, the level (mean fluorescence intensity [MFI]) of GzmB expression in individual NKT-like, CD4, and CD8 T cells was also significantly lower in CI than in UI (Fig. 4A to F). The frequencies of GzmB-producing CD8 T cells were significantly positively correlated with the days post-disease onset in CI (*r*^2^ = 0.139, *P* = 0.047, Fig. S2B). A tendency of increase in the frequencies of GzmB-producing CD4 T cells and NKT-like cells over time was also observed in CI (Fig. S2B). We did not observe significant differences in GzmB expression in NK cells (Fig. S5). Perforin expression was not different for all analyzed cell populations between CI and UI (Fig. S5). We also examined the production of inflammatory cytokines such as gamma interferon (IFN-γ), IL-6, and granulocyte-macrophage colony-stimulating factor (GM-CSF) by T cells, NK cells, and NKT-like cells in CI, since a previous study demonstrated that significant numbers of T cells produce these cytokines during the acute phase of COVID-19 (21). Although certain increases in the frequencies of CD4 and CD8 T cells producing IFN-γ, IL-6, and GM-CSF were observed in CI compared to UI (Fig. 4C and E), these differences were not statistically significant and appeared to be affected by two outliers in the CI group who had profound numbers of cytokine-producing T cells (Fig. S5B and C). No significant differences in frequencies of IFN-γ-, IL-6-, and GM-CSF-producing NK and NKT-like cells were observed between CI and UI (Fig. S5A and F).

10.1128/mBio.00085-21.5 FIG S5 Characterization of cytotoxic and cytokine profiles of T, NK, and NKT-like cells in the PBMCs of individuals recovering from COVID-19. (A) The percentages of perforin-, IFN-γ-, IL-6-, and GM-CSF-producing NKT-like cells and MFI (geometric mean) of perforin expression of NKT-like cells in the blood of UI (*n* = 21) and CI (*n* = 30) were analyzed by flow cytometry. (B) The percentages of perforin-, IFN-γ-, IL-6-, and GM-CSF-producing CD8 T cells and MFI (geometric mean) of perforin expression of CD8 T cells in the blood of UI (*n* = 21) and CI (*n* = 30) were analyzed by flow cytometry. (C) The percentages of perforin-, IFN-γ-, IL-6-, and GM-CSF-producing CD4 T cells and MFI (geometric mean) of perforin expression of CD4 T cells in the blood of UI (*n* = 21) and CI (*n* = 30) were analyzed by flow cytometry. (D) The fold changes of the percentages (mean) of granzyme B-, perforin-, IFN-γ-, IL-6-, and GM-CSF-producing NK cells in the blood of CI compared to those of UI are depicted by radar plots. (E) The percentages and MFI (geometric mean) of granzyme B expression of NK cells in the blood of UI (*n* = 16) and CI (*n* = 25) were analyzed by flow cytometry. (F) The percentages of perforin-, IFN-γ-, IL-6-, and GM-CSF-producing NK cells and MFI (geometric mean) of perforin expression of NK cells in the blood of UI (*n* = 21) and CI (*n* = 30) were analyzed by flow cytometry. CI, COVID-19-convalescent individuals; UI, SARS-CoV-2-unexposed individuals; ns, not significant (*P* > 0.05). Download FIG S5, TIF file, 0.6 MB. Copyright © 2021 Liu et al. 2021 Liu et al. https://creativecommons.org/licenses/by/4.0/ This content is distributed under the terms of the Creative Commons Attribution 4.0 International license.

**FIG 5:**
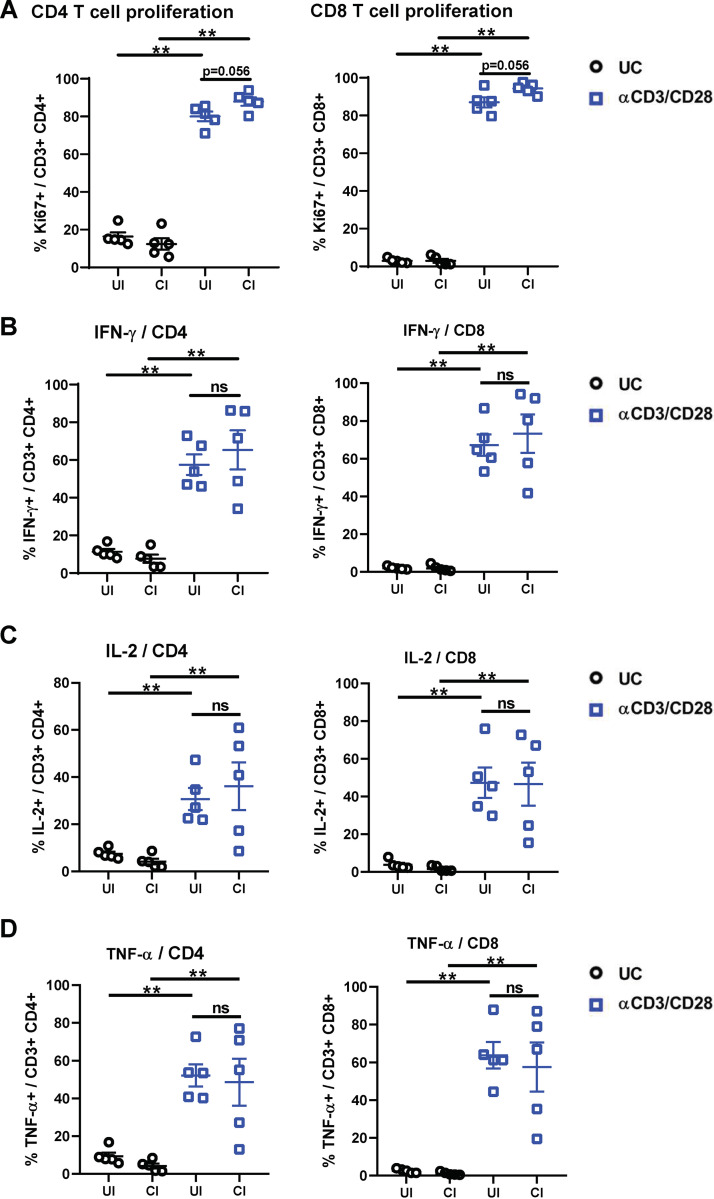
Characterization of the effector function of CD4 and CD8 T cells in the PBMCs of individuals recovering from COVID-19. PBMCs of UI (*n* = 5) and CI (*n* = 5) were either stimulated with anti-CD3 and anti-CD28 antibodies (αCD3/CD28) or left unstimulated (UC) and cultured for 5 days. The percentages of Ki67 (A)-, IFN-γ (B)-, IL-2 (C)-, and TNF-α (D)-positive CD4 (left) and CD8 (right) T cells were analyzed by flow cytometry. CI, COVID-19-convalescent individuals; UI, SARS-CoV-2-unexposed individuals. Statistically significant differences are indicated by asterisks (*, <0.05; **, <0.01; nonparametric Mann-Whitney test).

To further characterize effector functions of T cells in response to TCR stimulation, PBMCs from 5 CI and 5 UI were stimulated with anti-CD3/anti-CD28 for 5 days and were examined for cell proliferation (Ki67) and effector cytokine expression (IFN-γ, IL-2, and tumor necrosis factor alpha [TNF-α]). Compared to unstimulated cells, anti-CD3/anti-CD28 stimulation induced expected increases of Ki67 expression, as well as IFN-γ, IL-2, and TNF-α production by CD4 and CD8 T cells in both CI and UI (Fig. 5). No significant differences in effector cytokine production or proliferation of CD4 and CD8 T cells were observed between the groups (Fig. 5B to D).

Taken together, these results indicate that there is a long-term suppression of the cytotoxic potential of T cells after resolving SARS-CoV-2 infection; however, general effector functions of T cells in COVID-19-convalescent individuals are maintained.

## DISCUSSION

Wuhan was the very first city hit by SARS-CoV-2. Accordingly, the patients who experienced the longest phase of convalescence following COVID-19 reside here. This enabled us to investigate the “immunological scar” left by SARS-CoV-2 on cellular immunity after recovery from the disease. Our results reveal that 2 to 4 months after resolved SARS-CoV-2 infection, most components of cellular immunity returned to normal. However, the previous SARS-CoV-2 infection could still be recognized during convalescent phase by diminished numbers of NKT-like cells and iNKT cells as well as increased DCs. CI show an immune environment prone to suppression, supported by the observation of significantly increased Treg frequencies and upregulation of TIM-3 expression on T cells. Accordingly, the cytotoxic potential, as represented by GzmB expression, of T cells and NKT-like cells was significantly suppressed in CI. Both CD4 and CD8 T cells of CI showed increased cell proliferation and were fully capable of producing effector cytokines in response to TCR stimulation, suggesting the effector function of T cells is not compromised in CI.

Unexpectedly, our study revealed profound changes of NKT cells in the convalescent phase of COVID-19. NKT cells are a small but important subset of T lymphocytes that regulate immune responses in the context of infection, cancer, and autoimmunity (22). NKT cells can promote cell-mediated immunity to tumors and pathogens, yet they can also suppress the cell-mediated immunity associated with autoimmune diseases and are involved in the pathogenesis of many inflammatory disorders (23). NKT-like cells were shown to be cytotoxic toward lung epithelial cells and involved in the immunopathogenesis of pulmonary disease (24). It remains unclear by which means NKT cells carry out such opposing functions. The existence of functionally distinct NKT cell subsets may provide a rational explanation: so far, 3 NKT cell subsets, including classical NKT cells (iNKT cells), nonclassical NKT cells, and NKT-like cells, each expressing different TCRs, have been described (25). These cells are activated by lipid antigens linked to nonpolymorphic CD1 molecules and/or proinflammatory cytokines generated during infection and significantly contribute to the onset of infectious or autoimmune diseases (26). Reduced numbers of iNKT cells among PBMCs appear to correlate with the activity of systemic lupus erythematosus (SLE) disease (27). Selective loss of iNKT cells has also been reported during acute lymphocytic choriomeningitis virus (LCMV) infections (28), as well as in chronic HIV and hepatitis C virus (HCV) infection (29–31). Interestingly, subsequent long-term loss of iNKT cells during the convalescent phase following acute LCMV infection of mice has also been reported (32). It is believed that the reduction in iNKT cells at these late stages postinfection occurred by activation-induced cell death, since concomitant with the decrease in iNKT cells was an increase in the frequency of annexin V^+^ iNKT cells (32). Highly similar to the observation in acute LCMV infections of mice, we also demonstrate a selective long-term loss of iNKT and NKT-like cells 3 months after recovering from an acute SARS-CoV-2 infection. The observation of increases in the frequency of annexin V^+^ NKT-like cells in convalescent individuals suggests that the reduction of these cells may also occur by activation-induced cell death. Recent studies have reported that during acute SARS-CoV-2 infection, NKT-like cells showed a significant increase in GzmB and perforin production (33), as well as a decrease in numbers in severe COVID-19 cases (34), suggesting these cells are highly activated during the acute phase. A very recent study has also demonstrated the expansion of NKT CD160 cluster in moderate but not severe COVID-19 patients, which was believed to promote rapid control of the disease through direct cytotoxicity as well as mediating the antibody-dependent cell-mediated cytotoxicity effect (35). Taken together, these data suggest that the activation and subsequent long-term loss of NKT and NKT-like cells during COVID-19 are a normal component of the host’s antiviral immune response.

Comparative analyses in pneumonia induced by other microbiological origins, such as influenza A virus (IAV), will be informative to better gauge the role of NKT cells in SARS-CoV-2 infection. Many studies have demonstrated that iNKT cells contribute greatly to IAV immunity, as mice lacking iNKT cells show greater susceptibility (survival, enhanced inflammation) to IAV infections than iNKT cell-intact mice (36–39). iNKT cells could limit IAV replication through different mechanisms, including (i) reducing the suppressive capacity of myeloid-derived suppressor cells that inhibit IAV-specific CD8^+^ T cell response, (ii) activating lung-resident NK cells, and (iii) directly lysing IAV-infected monocytes (40). In addition, NKT cells also participate in the process of the resolution of inflammation, including tissue repair and regeneration. iNKT cells produce large amounts of tissue protective cytokine IL-22 during the early course of IAV infection (41). IL-22 does not affect viral loads but is critical for recovery of normal lung function and architecture after influenza virus infection (42, 43). IL-22 also reduces lung inflammation and protects against secondary bacterial infection during IAV infection (43). These findings are particularly relevant in the context of severe COVID-19 with ARDS, indicating NKT cells could also play a beneficial role in the context of SARS-CoV-2 infection. Actually, it has been reported that the percentage of NKT-like cells in the peripheral blood is significantly lower in the severe COVID-19 group than in the nonsevere one and is positively correlated with the pressure of arterial oxygen/fraction of inspired oxygen (PaO_2_/FiO_2_) ratio in patients (44). In addition, the activation marker CD69 expression on iNKT cells of COVID-19 patients on admission was also found to be positively correlated with the PaO_2_/FiO_2_ ratio and is believed to be predictive of clinical course and disease severity in severe COVID-19 (45). Altogether, these findings should encourage further studies on characterizing the mechanisms that regulate functions of NKT cells following SARS-CoV-2 infection and the potential of targeting NKT cells for immune intervention strategies.

In summary, we characterized the long-term impact of SARS-CoV-2 infection on the immune system and provide comprehensive picture of cellular immunity of a convalescent COVID-19 patient cohort with the longest recovery time. The overall alterations affecting cellular immunity observed in this study suggest that the immune system in convalescent individuals is going through a phase of restoring homeostasis after being highly activated during the acute phase of SARS-CoV-2 infection.

## MATERIALS AND METHODS

### Subjects.

Thirty convalescent individuals who resolved their SARS-CoV-2 infection and a matched group comprising 21 SARS-CoV-2-unexposed individuals were recruited at the Department of Infectious Diseases, Union Hospital, Tongji Medical College, Huazhong University of Science and Technology, from May to June 2020. The diagnosis of COVID-19 was based on the Guidelines for Diagnosis and Treatment of Corona Virus Disease 2019 issued by the National Health Commission of China (7th edition, http://www.chinacdc.cn/jkzt/crb/zl/szkb_11803/jszl_11815/202003/t20200305_214142.html). Informed written consent was obtained from each patient, and the study protocol was approved by the local medical ethics committee of Union Hospital, Tongji Medical College, Huazhong University of Science and Technology, in accordance with the guidelines of the Declaration of Helsinki (2020IEC-J-587). Invariant NKT cell analysis was performed in a German cohort which has 19 CI and 6 UI recruited at the Department of Gastroenterology, Hepatology and Infectious Diseases, Heinrich Heine University and University Hospital of Duesseldorf. Written informed consent was given by each included individual, and the study was approved by the ethics committee of the medical faculty of the Heinrich Heine University, Düsseldorf, Germany (study number: 5350).

### Preparation of PBMCs.

Peripheral blood mononuclear cells (PBMCs) of SARS-CoV-2-unexposed individuals and convalescent patients were isolated using Ficoll density gradient centrifugation (DAKEWE Biotech, Beijing, China) and were rapidly assessed by flow cytometry analysis without intermittent cryoconservation.

### Flow cytometry.

Surface and intracellular staining for flow cytometry analysis was performed as described previously (46, 47). For surface staining, cells were incubated with relevant fluorochrome-labeled antibodies for 30 min at 4°C in the dark. For intracellular cytokine staining, cells were fixed and permeabilized using the Intracellular Fixation & Permeabilization buffer set (Invitrogen, USA) and stained with allophycocyanin (APC)-anti-IFN-γ, peridinin chlorophyll protein (PerCP)-Cy5.5-anti-IL-2, or fluorescein isothiocyanate (FITC)-anti-TNF-α (BD Biosciences, USA). Freshly isolated cells were used for all assays. Approximately 100,000 PBMCs were acquired for each sample using a BD FACS Canto II flow cytometer. Data analysis was performed using FlowJo software V10.0.7 (Tree Star, Ashland, OR, USA). Cell debris and dead cells were excluded from the analysis based on scatter signals and Fixable Viability Dye eFluor 506.

### Analysis of effector T cell responses.

PBMCs were resuspended in complete medium (RPMI 1640 containing 10% fetal calf serum, 100 U/ml penicillin, 100 μg/ml streptomycin, and 100 μM 4-[2-hydroxyethyl]-1-piperazine ethanesulfonic acid buffer) and stimulated with anti-CD3 (1 μg/ml; BD Biosciences, USA), anti-CD28 (1 μg/ml; BD Biosciences, USA), and recombinant interleukin-2 (20 U/ml; Hoffmann-La Roche, Italy). Fresh medium containing IL-2 was added every 72 h. On day 5, brefeldin A (BD Biosciences, San Diego, CA) was added to the medium for 6 h. Cells were washed and tested for Ki67 expression and secretion of IFN-γ, IL-2, and TNF-α by intracellular cytokine staining and subsequent flow cytometry analyses.

### Statistical analysis.

Statistical analyses were performed using the SPSS statistical software package (version 22.0; SPSS Inc., Chicago, IL, USA). The Shapiro-Wilk method was used to test for normality. Mann-Whitney *t* test, Pearson product-moment correlation coefficient, and Fisher’s exact test were used where appropriate. All reported *P* values were two-sided, and a *P* value less than 0.05 was considered statistically significant.

## References

[1] Long QX, Tang XJ, Shi QL, Li Q, Deng HJ, Yuan J, Hu JL, Xu W, Zhang Y, Lv FJ, Su K, Zhang F, Gong J, Wu B, Liu XM, Li JJ, Qiu JF, Chen J, Huang AL. 2020. Clinical and immunological assessment of asymptomatic SARS-CoV-2 infections. Nat Med 26:1200–1204. 10.1038/s41591-020-0965-6. 32555424

[2] Guan WJ, Ni ZY, Hu Y, Liang WH, Ou CQ, He JX, Liu L, Shan H, Lei CL, Hui DSC, Du B, Li LJ, Zeng G, Yuen KY, Chen RC, Tang CL, Wang T, Chen PY, Xiang J, Li SY, Wang JL, Liang ZJ, Peng YX, Wei L, Liu Y, Hu YH, Peng P, Wang JM, Liu JY, Chen Z, Li G, Zheng ZJ, Qiu SQ, Luo J, Ye CJ, Zhu SY, Zhong NS, China Medical Treatment Expert Group for Covid-19. 2020. Clinical characteristics of coronavirus disease 2019 in China. N Engl J Med 382:1708–1720. 10.1056/NEJMoa2002032. 32109013 PMC7092819

[3] Puelles VG, Lutgehetmann M, Lindenmeyer MT, Sperhake JP, Wong MN, Allweiss L, Chilla S, Heinemann A, Wanner N, Liu S, Braun F, Lu S, Pfefferle S, Schroder AS, Edler C, Gross O, Glatzel M, Wichmann D, Wiech T, Kluge S, Pueschel K, Aepfelbacher M, Huber TB. 2020. Multiorgan and renal tropism of SARS-CoV-2. N Engl J Med 383:590–592. 10.1056/NEJMc2011400. 32402155 PMC7240771

[4] Asadi-Pooya AA, Simani L. 2020. Central nervous system manifestations of COVID-19: a systematic review. J Neurol Sci 413:116832. 10.1016/j.jns.2020.116832. 32299017 PMC7151535

[5] Huang C, Wang Y, Li X, Ren L, Zhao J, Hu Y, Zhang L, Fan G, Xu J, Gu X, Cheng Z, Yu T, Xia J, Wei Y, Wu W, Xie X, Yin W, Li H, Liu M, Xiao Y, Gao H, Guo L, Xie J, Wang G, Jiang R, Gao Z, Jin Q, Wang J, Cao B. 2020. Clinical features of patients infected with 2019 novel coronavirus in Wuhan, China. Lancet 395:497–506. 10.1016/S0140-6736(20)30183-5. 31986264 PMC7159299

[6] Chan JF, Yuan S, Kok KH, To KK, Chu H, Yang J, Xing F, Liu J, Yip CC, Poon RW, Tsoi HW, Lo SK, Chan KH, Poon VK, Chan WM, Ip JD, Cai JP, Cheng VC, Chen H, Hui CK, Yuen KY. 2020. A familial cluster of pneumonia associated with the 2019 novel coronavirus indicating person-to-person transmission: a study of a family cluster. Lancet 395:514–523. 10.1016/S0140-6736(20)30154-9. 31986261 PMC7159286

[7] Yang X, Yu Y, Xu J, Shu H, Xia J, Liu H, Wu Y, Zhang L, Yu Z, Fang M, Yu T, Wang Y, Pan S, Zou X, Yuan S, Shang Y. 2020. Clinical course and outcomes of critically ill patients with SARS-CoV-2 pneumonia in Wuhan, China: a single-centered, retrospective, observational study. Lancet Respir Med 8:475–481. 10.1016/S2213-2600(20)30079-5. 32105632 PMC7102538

[8] Vabret N, Britton GJ, Gruber C, Hegde S, Kim J, Kuksin M, Levantovsky R, Malle L, Moreira A, Park MD, Pia L, Risson E, Saffern M, Salomé B, Esai Selvan M, Spindler MP, Tan J, van der Heide V, Gregory JK, Alexandropoulos K, Bhardwaj N, Brown BD, Greenbaum B, Gümüş ZH, Homann D, Horowitz A, Kamphorst AO, Curotto de Lafaille MA, Mehandru S, Merad M, Samstein RM, Sinai Immunology Review Project. 2020. Immunology of COVID-19: current state of the science. Immunity 52:910–941. 10.1016/j.immuni.2020.05.002. 32505227 PMC7200337

[9] Liu J, Li S, Liu J, Liang B, Wang X, Wang H, Li W, Tong Q, Yi J, Zhao L, Xiong L, Guo C, Tian J, Luo J, Yao J, Pang R, Shen H, Peng C, Liu T, Zhang Q, Wu J, Xu L, Lu S, Wang B, Weng Z, Han C, Zhu H, Zhou R, Zhou H, Chen X, Ye P, Zhu B, Wang L, Zhou W, He S, He Y, Jie S, Wei P, Zhang J, Lu Y, Wang W, Zhang L, Li L, Zhou F, Wang J, Dittmer U, Lu M, Hu Y, Yang D, Zheng X. 2020. Longitudinal characteristics of lymphocyte responses and cytokine profiles in the peripheral blood of SARS-CoV-2 infected patients. EBioMedicine 55:102763. 10.1016/j.ebiom.2020.102763. 32361250 PMC7165294

[10] Wang D, Hu B, Hu C, Zhu F, Liu X, Zhang J, Wang B, Xiang H, Cheng Z, Xiong Y, Zhao Y, Li Y, Wang X, Peng Z. 2020. Clinical characteristics of 138 hospitalized patients with 2019 novel coronavirus-infected pneumonia in Wuhan, China. JAMA 323:1061–1069. 10.1001/jama.2020.1585. 32031570 PMC7042881

[11] Mehta P, McAuley DF, Brown M, Sanchez E, Tattersall RS, Manson JJ, HLH Across Speciality Collaboration, UK. 2020. COVID-19: consider cytokine storm syndromes and immunosuppression. Lancet 395:1033–1034. 10.1016/S0140-6736(20)30628-0. 32192578 PMC7270045

[12] Moore JB, June CH. 2020. Cytokine release syndrome in severe COVID-19. Science 368:473–474. 10.1126/science.abb8925. 32303591

[13] Zheng HY, Zhang M, Yang CX, Zhang N, Wang XC, Yang XP, Dong XQ, Zheng YT. 2020. Elevated exhaustion levels and reduced functional diversity of T cells in peripheral blood may predict severe progression in COVID-19 patients. Cell Mol Immunol 17:541–543. 10.1038/s41423-020-0401-3. 32203186 PMC7091621

[14] Diao B, Wang C, Tan Y, Chen X, Liu Y, Ning L, Chen L, Li M, Liu Y, Wang G, Yuan Z, Feng Z, Zhang Y, Wu Y, Chen Y. 2020. Reduction and functional exhaustion of T cells in patients with coronavirus disease 2019 (COVID-19). Front Immunol 11:827. 10.3389/fimmu.2020.00827. 32425950 PMC7205903

[15] Westmeier J, Paniskaki K, Karakose Z, Werner T, Sutter K, Dolff S, Overbeck M, Limmer A, Liu J, Zheng X, Brenner T, Berger MM, Witzke O, Trilling M, Lu M, Yang D, Babel N, Westhoff T, Dittmer U, Zelinskyy G. 2020. Impaired cytotoxic CD8(+) T cell response in elderly COVID-19 patients. mBio 11:e02805-20. 10.1128/mBio.02805-20. 32948688 PMC7502863

[16] Alfei F, Kanev K, Hofmann M, Wu M, Ghoneim HE, Roelli P, Utzschneider DT, von Hoesslin M, Cullen JG, Fan Y, Eisenberg V, Wohlleber D, Steiger K, Merkler D, Delorenzi M, Knolle PA, Cohen CJ, Thimme R, Youngblood B, Zehn D. 2019. TOX reinforces the phenotype and longevity of exhausted T cells in chronic viral infection. Nature 571:265–269. 10.1038/s41586-019-1326-9. 31207605

[17] Khan O, Giles JR, McDonald S, Manne S, Ngiow SF, Patel KP, Werner MT, Huang AC, Alexander KA, Wu JE, Attanasio J, Yan P, George SM, Bengsch B, Staupe RP, Donahue G, Xu W, Amaravadi RK, Xu X, Karakousis GC, Mitchell TC, Schuchter LM, Kaye J, Berger SL, Wherry EJ. 2019. TOX transcriptionally and epigenetically programs CD8(+) T cell exhaustion. Nature 571:211–218. 10.1038/s41586-019-1325-x. 31207603 PMC6713202

[18] Belkaid Y. 2008. Role of Foxp3-positive regulatory T cells during infection. Eur J Immunol 38:918–921. 10.1002/eji.200738120. 18395860 PMC4052573

[19] Zelinskyy G, Dietze KK, Husecken YP, Schimmer S, Nair S, Werner T, Gibbert K, Kershaw O, Gruber AD, Sparwasser T, Dittmer U. 2009. The regulatory T-cell response during acute retroviral infection is locally defined and controls the magnitude and duration of the virus-specific cytotoxic T-cell response. Blood 114:3199–3207. 10.1182/blood-2009-03-208736. 19671923

[20] Hasenkrug KJ, Chougnet CA, Dittmer U. 2018. Regulatory T cells in retroviral infections. PLoS Pathog 14:e1006776. 10.1371/journal.ppat.1006776. 29447279 PMC5814043

[21] Zhou Y, Fu B, Zheng X, Wang D, Zhao C, Qi Y, Sun R, Tian Z, Xu X, Wei H. 2020. Pathogenic T-cells and inflammatory monocytes incite inflammatory storms in severe COVID-19 patients. Natl Sci Rev 7:998–1002. 10.1093/nsr/nwaa041. PMC7108005 34676125

[22] Slauenwhite D, Johnston B. 2015. Regulation of NKT cell localization in homeostasis and infection. Front Immunol 6:255. 10.3389/fimmu.2015.00255. 26074921 PMC4445310

[23] Godfrey DI, Stankovic S, Baxter AG. 2010. Raising the NKT cell family. Nat Immunol 11:197–206. 10.1038/ni.1841. 20139988

[24] Hodge G, Hodge S. 2016. Steroid resistant CD8(+)CD28(null) NKT-like pro-inflammatory cytotoxic cells in chronic obstructive pulmonary disease. Front Immunol 7:617. 10.3389/fimmu.2016.00617. 28066427 PMC5165019

[25] Godfrey DI, MacDonald HR, Kronenberg M, Smyth MJ, Van Kaer L. 2004. NKT cells: what’s in a name? Nat Rev Immunol 4:231–237. 10.1038/nri1309. 15039760

[26] Torina A, Guggino G, La Manna MP, Sireci G. 2018. The Janus face of NKT cell function in autoimmunity and infectious diseases. Int J Mol Sci 19:440. 10.3390/ijms19020440. PMC5855662 29389901

[27] Cho YN, Kee SJ, Lee SJ, Seo SR, Kim TJ, Lee SS, Kim MS, Lee WW, Yoo DH, Kim N, Park YW. 2011. Numerical and functional deficiencies of natural killer T cells in systemic lupus erythematosus: their deficiency related to disease activity. Rheumatology (Oxford) 50:1054–1063. 10.1093/rheumatology/keq457. 21278064

[28] Hobbs JA, Cho S, Roberts TJ, Sriram V, Zhang J, Xu M, Brutkiewicz RR. 2001. Selective loss of natural killer T cells by apoptosis following infection with lymphocytic choriomeningitis virus. J Virol 75:10746–10754. 10.1128/JVI.75.22.10746-10754.2001. 11602716 PMC114656

[29] Sandberg JK, Fast NM, Palacios EH, Fennelly G, Dobroszycki J, Palumbo P, Wiznia A, Grant RM, Bhardwaj N, Rosenberg MG, Nixon DF. 2002. Selective loss of innate CD4(+) V alpha 24 natural killer T cells in human immunodeficiency virus infection. J Virol 76:7528–7534. 10.1128/jvi.76.15.7528-7534.2002. 12097565 PMC136353

[30] van der Vliet HJ, von Blomberg BM, Hazenberg MD, Nishi N, Otto SA, van Benthem BH, Prins M, Claessen FA, van den Eertwegh AJ, Giaccone G, Miedema F, Scheper RJ, Pinedo HM. 2002. Selective decrease in circulating V alpha 24+V beta 11+ NKT cells during HIV type 1 infection. J Immunol 168:1490–1495. 10.4049/jimmunol.168.3.1490. 11801694

[31] Lucas M, Gadola S, Meier U, Young NT, Harcourt G, Karadimitris A, Coumi N, Brown D, Dusheiko G, Cerundolo V, Klenerman P. 2003. Frequency and phenotype of circulating Valpha24/Vbeta11 double-positive natural killer T cells during hepatitis C virus infection. J Virol 77:2251–2257. 10.1128/jvi.77.3.2251-2257.2003. 12525661 PMC140901

[32] Lin Y, Roberts TJ, Wang CR, Cho S, Brutkiewicz RR. 2005. Long-term loss of canonical NKT cells following an acute virus infection. Eur J Immunol 35:879–889. 10.1002/eji.200425495. 15724241

[33] Ahmadi P, Hartjen P, Kohsar M, Kummer S, Schmiedel S, Bockmann JH, Fathi A, Huber S, Haag F, Schulze Zur Wiesch J. 2020. Defining the CD39/CD73 axis in SARS-CoV-2 infection: the CD73(-) phenotype identifies polyfunctional cytotoxic lymphocytes. Cells 9:1750. 10.3390/cells9081750. PMC7464076 32707842

[34] Odak I, Barros-Martins J, Bosnjak B, Stahl K, David S, Wiesner O, Busch M, Hoeper MM, Pink I, Welte T, Cornberg M, Stoll M, Goudeva L, Blasczyk R, Ganser A, Prinz I, Forster R, Koenecke C, Schultze-Florey CR. 2020. Reappearance of effector T cells is associated with recovery from COVID-19. EBioMedicine 57:102885. 10.1016/j.ebiom.2020.102885. 32650275 PMC7338277

[35] Zhang JY, Wang XM, Xing X, Xu Z, Zhang C, Song JW, Fan X, Xia P, Fu JL, Wang SY, Xu RN, Dai XP, Shi L, Huang L, Jiang TJ, Shi M, Zhang Y, Zumla A, Maeurer M, Bai F, Wang FS. 2020. Single-cell landscape of immunological responses in patients with COVID-19. Nat Immunol 21:1107–1118. 10.1038/s41590-020-0762-x. 32788748

[36] De Santo C, Salio M, Masri SH, Lee LY, Dong T, Speak AO, Porubsky S, Booth S, Veerapen N, Besra GS, Grone HJ, Platt FM, Zambon M, Cerundolo V. 2008. Invariant NKT cells reduce the immunosuppressive activity of influenza A virus-induced myeloid-derived suppressor cells in mice and humans. J Clin Invest 118:4036–4048. 10.1172/JCI36264. 19033672 PMC2582442

[37] Ishikawa H, Tanaka K, Kutsukake E, Fukui T, Sasaki H, Hata A, Noda S, Matsumoto T. 2010. IFN-gamma production downstream of NKT cell activation in mice infected with influenza virus enhances the cytolytic activities of both NK cells and viral antigen-specific CD8+ T cells. Virology 407:325–332. 10.1016/j.virol.2010.08.030. 20855097

[38] Kok WL, Denney L, Benam K, Cole S, Clelland C, McMichael AJ, Ho LP. 2012. Pivotal advance: invariant NKT cells reduce accumulation of inflammatory monocytes in the lungs and decrease immune-pathology during severe influenza A virus infection. J Leukoc Biol 91:357–368. 10.1189/jlb.0411184. 22003207

[39] Paget C, Ivanov S, Fontaine J, Blanc F, Pichavant M, Renneson J, Bialecki E, Pothlichet J, Vendeville C, Barba-Spaeth G, Huerre M-R, Faveeuw C, Si-Tahar M, Trottein F. 2011. Potential role of invariant NKT cells in the control of pulmonary inflammation and CD8+ T cell response during acute influenza A virus H3N2 pneumonia. J Immunol 186:5590–5602. 10.4049/jimmunol.1002348. 21490153

[40] Driver JP, de Carvalho Madrid DM, Gu W, Artiaga BL, Richt JA. 2020. Modulation of immune responses to influenza A virus vaccines by natural killer T cells. Front Immunol 11:2172. 10.3389/fimmu.2020.02172. 33193296 PMC7606973

[41] Paget C, Ivanov S, Fontaine J, Renneson J, Blanc F, Pichavant M, Dumoutier L, Ryffel B, Renauld JC, Gosset P, Gosset P, Si-Tahar M, Faveeuw C, Trottein F. 2012. Interleukin-22 is produced by invariant natural killer T lymphocytes during influenza A virus infection: potential role in protection against lung epithelial damages. J Biol Chem 287:8816–8829. 10.1074/jbc.M111.304758. 22294696 PMC3308738

[42] Pociask DA, Scheller EV, Mandalapu S, McHugh KJ, Enelow RI, Fattman CL, Kolls JK, Alcorn JF. 2013. IL-22 is essential for lung epithelial repair following influenza infection. Am J Pathol 182:1286–1296. 10.1016/j.ajpath.2012.12.007. 23490254 PMC3620404

[43] Ivanov S, Renneson J, Fontaine J, Barthelemy A, Paget C, Fernandez EM, Blanc F, De Trez C, Van Maele L, Dumoutier L, Huerre MR, Eberl G, Si-Tahar M, Gosset P, Renauld JC, Sirard JC, Faveeuw C, Trottein F. 2013. Interleukin-22 reduces lung inflammation during influenza A virus infection and protects against secondary bacterial infection. J Virol 87:6911–6924. 10.1128/JVI.02943-12. 23596287 PMC3676141

[44] Zingaropoli MA, Perri V, Pasculli P, Cogliati Dezza F, Nijhawan P, Savelloni G, La Torre G, D’Agostino C, Mengoni F, Lichtner M, Ciardi MR, Mastroianni CM. 2021. Major reduction of NKT cells in patients with severe COVID-19 pneumonia. Clin Immunol 222:108630. 10.1016/j.clim.2020.108630. 33189887 PMC7661928

[45] Jouan Y, Guillon A, Gonzalez L, Perez Y, Boisseau C, Ehrmann S, Ferreira M, Daix T, Jeannet R, Francois B, Dequin PF, Si-Tahar M, Baranek T, Paget C. 2020. Phenotypical and functional alteration of unconventional T cells in severe COVID-19 patients. J Exp Med 217:e20200872. 10.1084/jem.20200872. 32886755 PMC7472174

[46] Liu J, Jiang M, Ma Z, Dietze KK, Zelinskyy G, Yang D, Dittmer U, Schlaak JF, Roggendorf M, Lu M. 2013. TLR1/2 ligand-stimulated mouse liver endothelial cells secrete IL-12 and trigger CD8+ T cell immunity in vitro. J Immunol 191:6178–6190. 10.4049/jimmunol.1301262. 24227786

[47] Wang Q, Pan W, Liu Y, Luo J, Zhu D, Lu Y, Feng X, Yang X, Dittmer U, Lu M, Yang D, Liu J. 2018. Hepatitis B virus-specific CD8+ T cells maintain functional exhaustion after antigen reexposure in an acute activation immune environment. Front Immunol 9:219. 10.3389/fimmu.2018.00219. 29483916 PMC5816053

