# Supplementary figures and images for: Analysis of the Long-Term Impact on Cellular Immunity in COVID-19-Recovered Individuals Reveals a Profound NKT Cell Impairment
Source: mBio. 2021 Apr 27;12(2):e00085-21. doi: 10.1128/mBio.00085-21 (PMC8092197; doi:10.1128/mBio.00085-21)

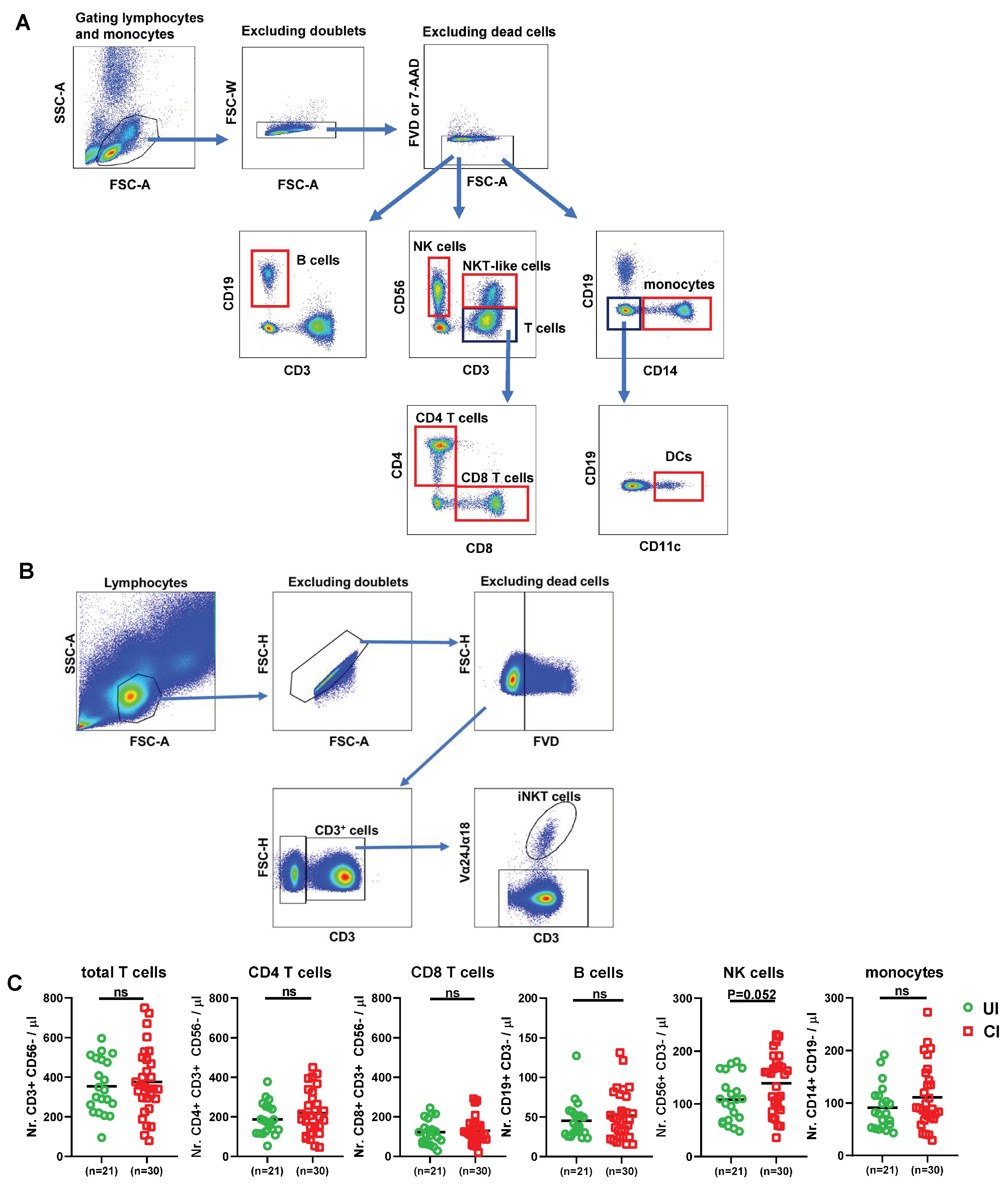

Supplement: FIG S1 [file mBio.00085-21-sf001.tif]

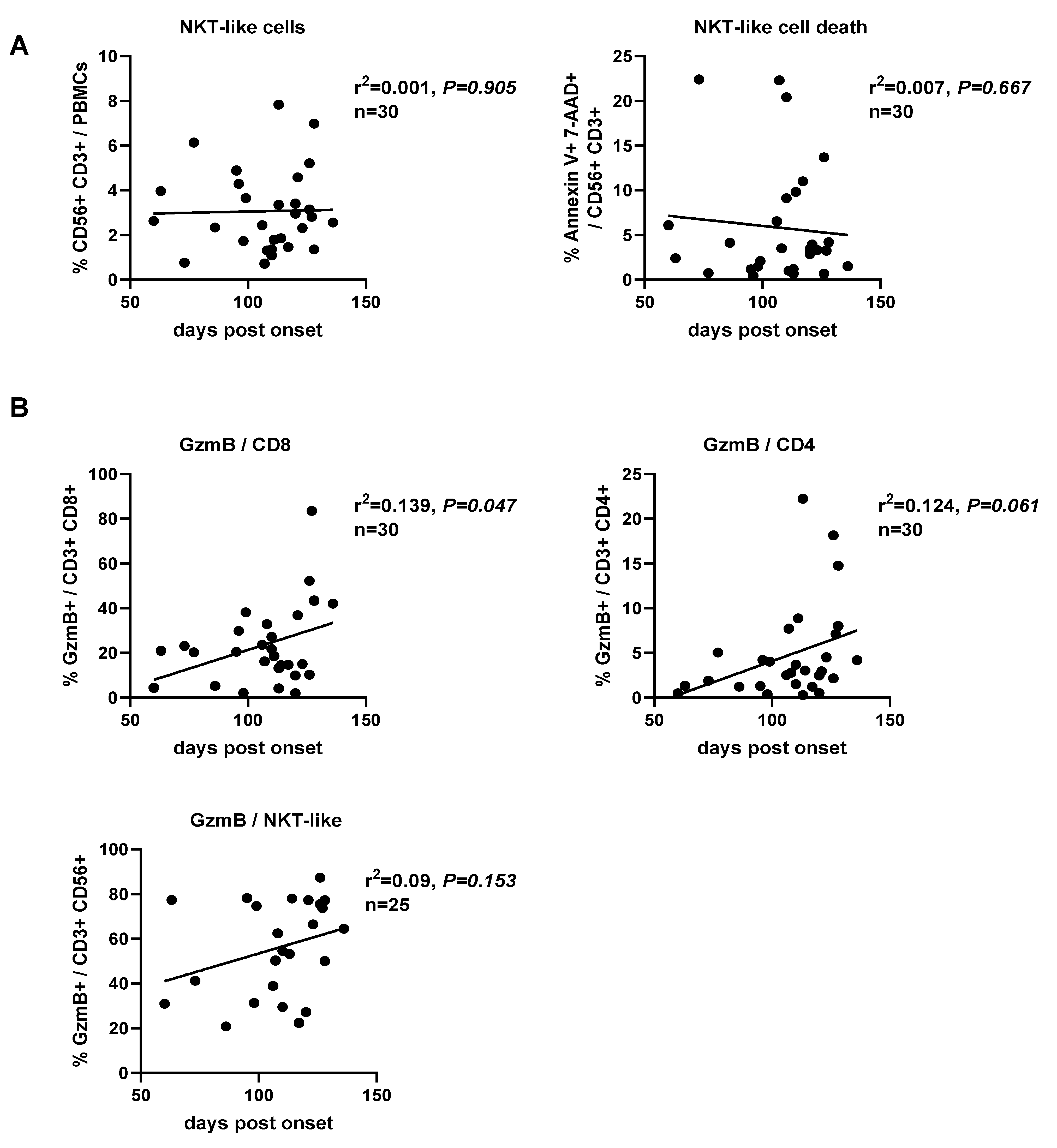

Supplement: FIG S2 [file mBio.00085-21-sf002.tif]

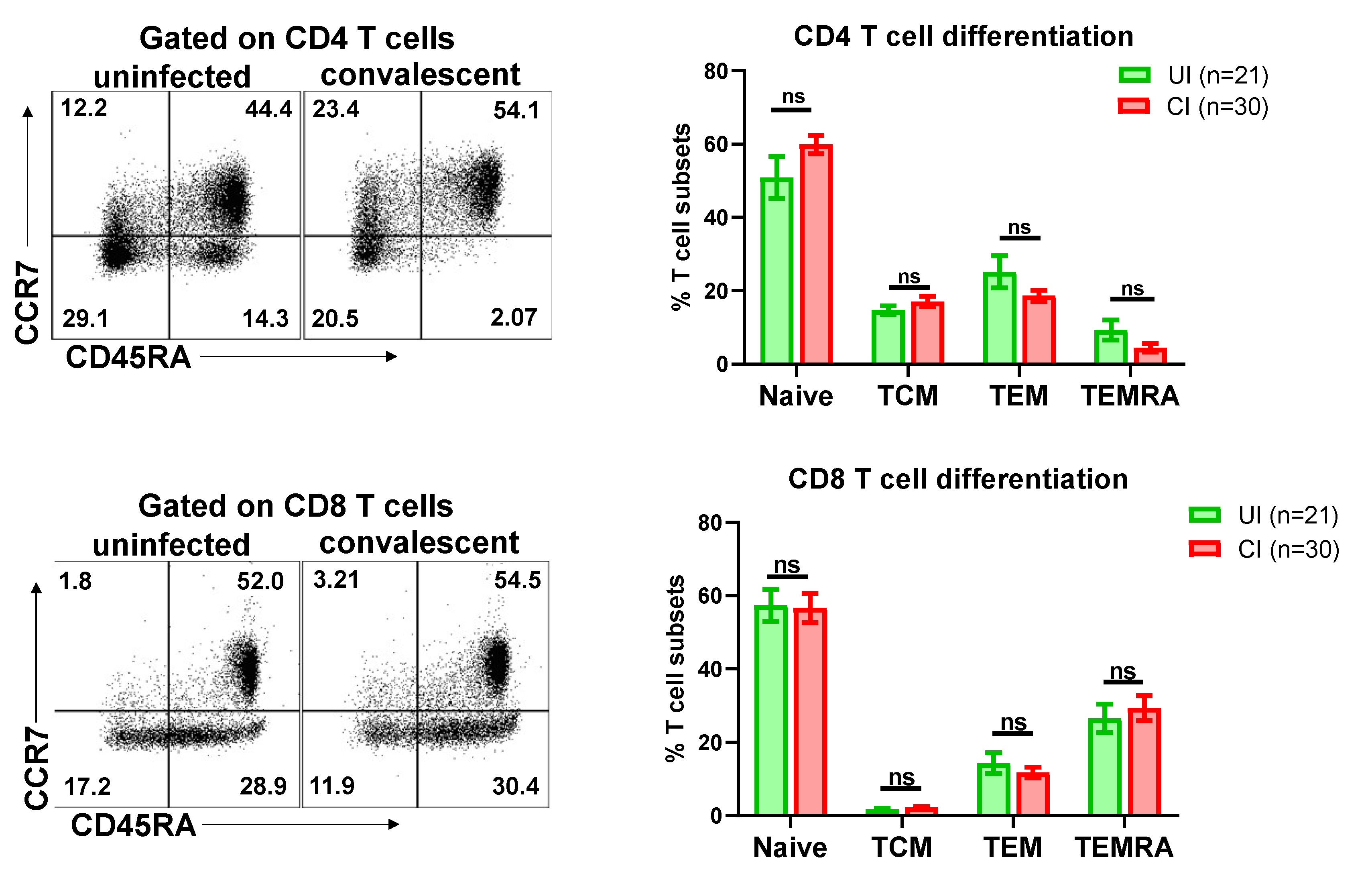

Supplement: FIG S3 [file mBio.00085-21-sf003.tif]

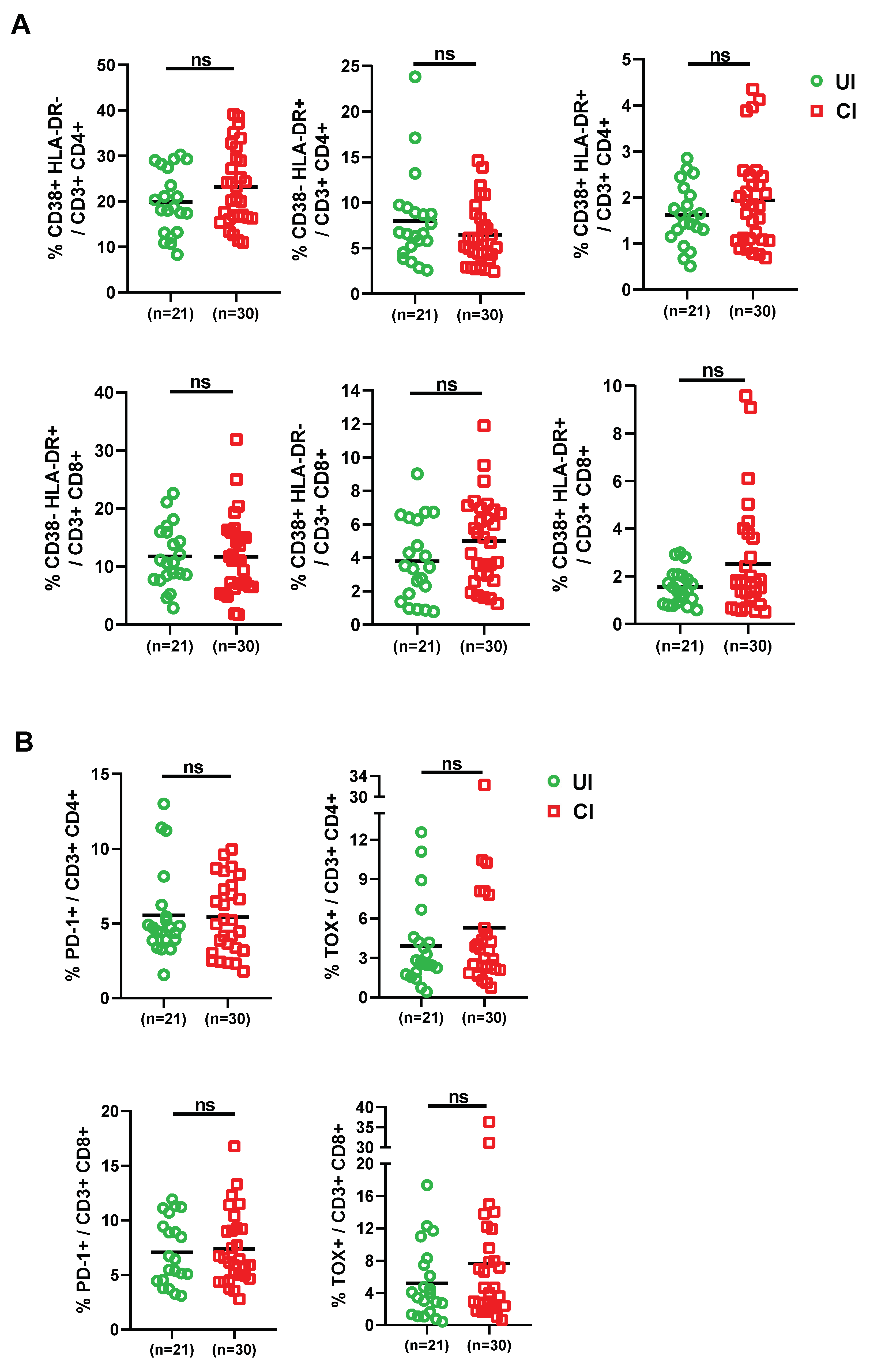

Supplement: FIG S4 [file mBio.00085-21-sf004.tif]

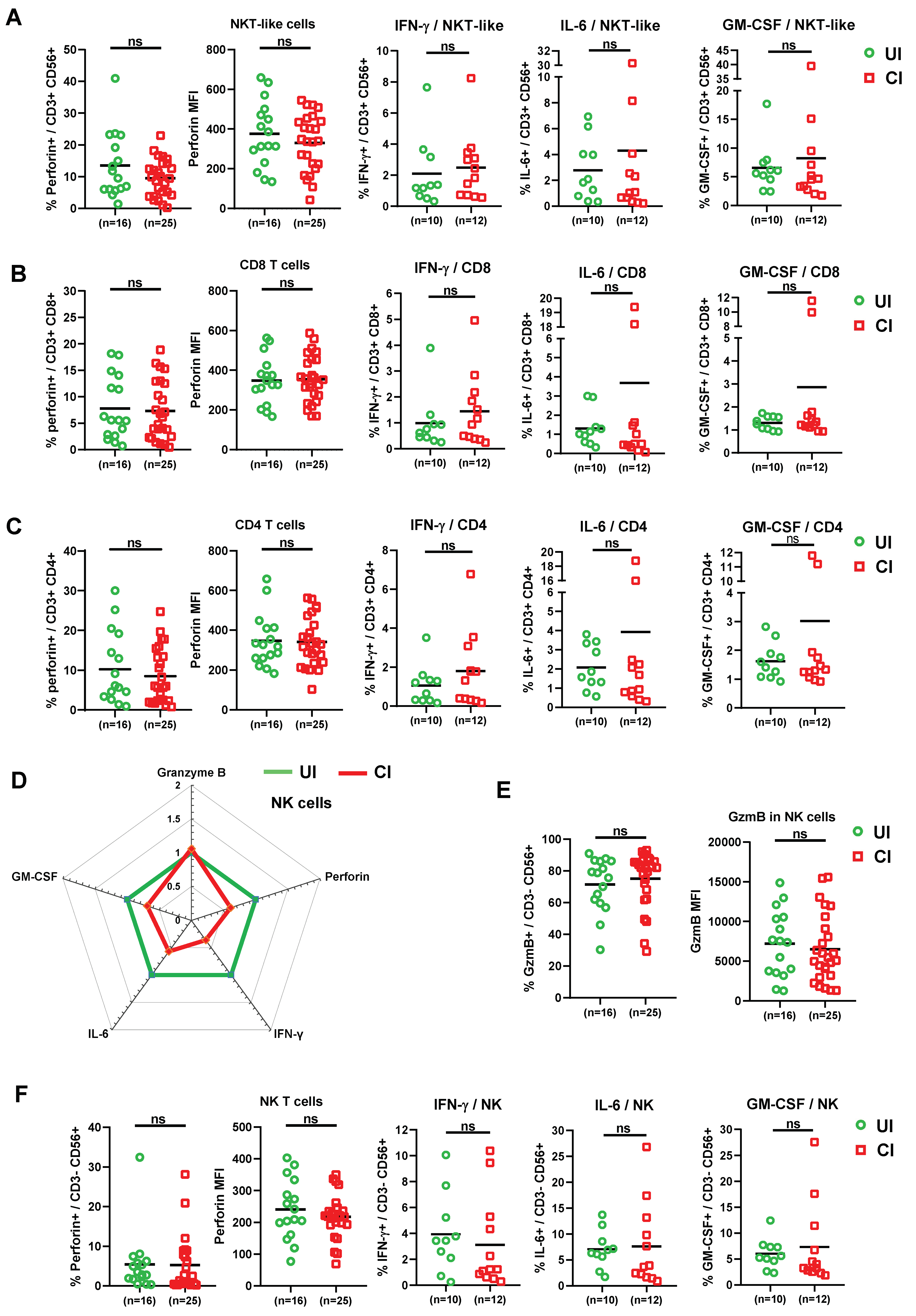

Supplement: FIG S5 [file mBio.00085-21-sf005.tif]
